# The SAGES scoring model leads to personalized treatment via the combination of TACE, TKIs and ICIs for unresectable hepatocellular carcinoma

**DOI:** 10.3389/fimmu.2026.1802427

**Published:** 2026-04-23

**Authors:** Hesheng Lin, Xueyao Wang, Jianhong Zhong, Ning Peng, Shaoping Liu, Fanjian Zeng, Wenfeng Li, Ze Su, Junjie Ou, Peisheng Wu, Hongbing Yao, Shuqun Li

**Affiliations:** 1 Department of Hepatobiliary and Pancreatic Surgery, Affiliated Hospital of Guilin Medical University, Guilin, China; 2 Department of Hepatobiliary and Pancreatic Surgery, Second Affiliated Hospital of Guilin Medical University, Guilin, China; 3 Department of Hepatobiliary Surgery, Guangxi Medical University Cancer Hospital, Nanning, China; 4 Department of Hepatobiliary Surgery, First Affiliated Hospital of Guangxi Medical University, Nanning, China; 5 Department of Hepatobiliary and Pancreatic Surgery, Eighth Affiliated Hospital of Guangxi Medical University, Guigang, China; 6 Department of Hepatobiliary Surgery, Wuzhou Red Cross Hospital, Wuzhou, China; 7 Department of Hepatobiliary and Pancreatic Surgery, First People’s Hospital of Yulin, Yulin, China; 8 Department of Hepatobiliary and Pancreatic Surgery, First People’s Hospital of Nanning, Nanning, China; 9 Department of Hepatobiliary Surgery, Wuzhou People’s Hospital, Wuzhou, China; 10 Department of Hepatobiliary and Pancreatic Surgery, First People’s Hospital of Qinzhou, Qinzhou, China

**Keywords:** immune checkpoint inhibitor, overall survival, progression-free survival, sages score, transarterial chemoembolization, tyrosine kinase inhibitor, unresectable hepatocellular carcinoma

## Abstract

**Background and aims:**

Transcatheter chemoembolization (TACE) combined with tyrosine kinase inhibitors (TKIs) and immune checkpoint inhibitors (ICIs) shows promising efficacy in treating unresectable hepatocellular carcinoma (uHCC), but the specific patient population that would benefit most from this regimen remains unclear. This study aims to evaluate the prognoses of uHCC patients receiving triple therapy and develop a practical prognostic scoring model to identify those with the best beneficial.

**Methods:**

This multicenter retrospective study enrolled 270 uHCC patients who received first-line triple therapy across 20 centers. These participants were divided into the training (n=190) and external validation (n=80) cohorts. Treatment response was assessed by the modified Response Evaluation Criteria in Solid Tumors (mRECIST), and safety was evaluated via treatment-related adverse events (TRAEs) using National Cancer Institute Common Terminology Criteria for Adverse Events version 5.0 (NCI-CTCAE v5.0). Cox proportional hazards regression was used to identify independent prognostic factors for overall survival, which were utilized to develop the SAGES score; Kaplan-Meier curves and area under the receiver operating characteristic curve (AUC) were employed to validate the model’s performance.

**Results:**

In the training cohort, the objective response rate was 47.9% and disease control rate was 63.2%. The median progression-free survival was 15.9 months, with 3-year overall survival and progression-free survival rates of 52.2% and 30.7%, respectively. Independent prognostic factors for poor overall survival included albumin-bilirubin grade 2–3, alpha-fetoprotein ≥400 ng/mL, maximum tumor size ≥8 cm, presence of extrahepatic metastasis, and absence of conversion surgery. Integrating these five factors, the SAGES score effectively stratified patients into low- (0–3 points), intermediate- (4–7 points), and high-risk (8–10 points) groups with significantly divergent survival outcomes in both cohorts (all p<0.001). The model exhibited robust discriminative ability, with AUCs of 0.78 in the training cohort and 0.75 in the validation cohort, outperforming individual prognostic factors.

**Conclusion:**

Triple therapy showed promising clinical outcomes and the SAGES score provides reliable prognostic stratification and facilitates personalized treatment decisions for uHCC patients receiving this triple regimen.

## Introduction

Hepatocellular carcinoma (HCC) remains a major global health challenge, threatening human well-being and exerting a substantial strain on healthcare infrastructures worldwide. As the sixth most common malignant tumor and the third leading cause of cancer-related deaths worldwide, HCC poses a severe threat to public health (1). Due to the lack of distinct early symptoms, approximately 70% of patients are discovered at an advanced stage, resulting in the loss of access to curative treatments (2).

As systemic therapies, including immune checkpoint inhibitors (ICIs) and molecularly targeted agents, have evolved at a rapid pace in recent years, the available treatments for unresectable hepatocellular carcinoma (uHCC) have significantly increased (3, 4). However, single-agent therapeutic approaches, such as transarterial chemoembolization (TACE), radiotherapy, molecularly targeted agents, and immunotherapies, have demonstrated limited therapy efficacy (5, 6). Recent advances in HCC management have shifted the paradigm from monotherapy to multimodal therapeutic regimens. To improve patient outcomes with uHCC, a variety of combination regimens have been created. For advanced HCC, combinatorial treatment involving tyrosine kinase inhibitors (TKIs) and ICIs has been linked to a marked improvement in patient prognosis. This combination has shown promising clinical results, with a median overall survival (OS) of approximately 20 months and an objective response rate (ORR) ranging from 35.0% to 76.7% (7–9). TACE is a successful local uHCC therapy technique and is widely used in Asia. Accumulating evidence has confirmed that the integration of TACE with systemic treatments has emerged as a promising therapeutic strategy. Prior research has indicated that combining TACE, lenvatinib, and PD-1 inhibitors enhances OS and progression-free survival (PFS) in patients with uHCC while preserving a favorable safety profile (10, 11). Li et al. demonstrated that combining TACE, lenvatinib and PD-1 inhibitors achieved an ORR of 60.9% according to the modified Response Evaluation Criteria in Solid Tumors (mRECIST) (12).

However, in real-world clinical practice, patients may receive different targeted agents or switch therapies due to multiple factors, such as treatment-related adverse events, economic burden, and drug availability. The GUIDANCE001 study, a large multicenter retrospective study on uHCC conversion therapy, also used a variety of TKIs and ICIs in the triple therapy group, and confirmed that the overall efficacy of triple therapy was significantly superior to TACE alone, which supports the clinical rationality of using multiple TKIs and ICIs in real-world research (13). Additionally, the precise subgroup of patients who derive the greatest benefit from triple therapy remains undefined. Therefore, we aimed to investigate the efficacy and safety of triple regimen of TACE combined with TKIs and ICIs for the management of uHCC. In this multicenter retrospective study, we evaluated the therapeutic outcomes of this triple therapy in uHCC patients and further developed and validated a predictive model to identify the population most likely to respond to treatment.

## Patients and methods

This multicenter retrospective study enrolled patients diagnosed with uHCC who were treated with first-line triple therapy (TACE + TKIs + ICIs) across 20 tertiary hospitals in China from January 1, 2019, to June 30, 2023. Study data were retrieved from the prospective registry database of the “Guangxi Liver Cancer Clinical Study Alliance (GUIDANCE)” curated by the Liver Cancer Committee of the Guangxi Anticancer Association, registered on ClinicalTrials.gov (Identifier: NCT06405321) (13–16). The research protocol adhered to the Declaration of Helsinki and was approved by ethics committees of all participating institutions (17). The study population was divided into a training cohort enrolled from multiple participating hospitals and an independent external validation cohort comprising patients from the remaining centers. We conducted a retrospective analysis of demographic characteristics, clinical parameters (including liver function profiles, tumor burden assessments, and other relevant clinical indices), laboratory results, imaging findings, and treatment response data.

The diagnosis of HCC was established based on the Chinese Guidelines for Liver Cancer, confirmed by either typical radiological manifestations or histopathological examination (18). All enrolled HCC patients were staged using the Barcelona Clinic Liver Cancer (BCLC) staging system (19). Tumor unresectability was evaluated and confirmed by a multidisciplinary team based on the following criteria (1): bilobar hepatic involvement that precluded R0 resection (2); inadequate surgical margins or insufficient future liver remnant volume (3); extrahepatic metastasis (EHM) or extrahepatic lymph node metastasis (LNM) (20).

The inclusion criteria for study enrollment were as follows (1): age between 18 and 75 years (2); Eastern Cooperative Oncology Group performance status (ECOG-PS) score of 0–1 (3); no prior systemic or locoregional treatment for HCC (4); uHCC with Child-Pugh class A or B liver function, and first-line triple therapy involving TACE combined with TKIs and ICIs. Exclusion criteria included (1): concurrent malignancies (2); incomplete clinical documentation (3); treatment interruption attributable to intolerance or non-adherence (4); HCC classified as BCLC stage A.

### TACE procedure

All TACE operations were performed by interventional radiologists with at least 10 years of clinical experience in accordance with Chinese clinical practice guidelines (18). A microcatheter (2.7 F) was selectively inserted into the arterial branch feeding the tumor under the supervision of digital subtraction angiography (DSA). This was followed by a gradual infusion of epirubicin (30–50 mg) combined with iodized oil (5–20 ml), with the total volume of emulsion adjusted according to three-dimensional tumor measurements obtained from preprocedural imaging. Subsequently, gelatin sponge particles (150-350μm) were administered to embolize the target vessels, thereby achieving sustained occlusion of the tumor blood supply. The endpoint of embolization was defined as stasis of blood flow in the tumor-feeding artery. Repeat TACE was performed on an on-demand basis. The decision was made by the multidisciplinary team based on residual tumor activity (enhanced lesion on contrast-enhanced CT/MRI) and liver function at 4–6 weeks after the last TACE session. The median number of TACE cycles was 3 (interquartile range, IQR, 2-4), and the total number of TACE sessions per patient was limited to fewer than 6.

### TKIs and ICIs

Patients received at least one TKI with the following dosing regimens: lenvatinib administered orally once daily at 8 mg for patients weighing <60 kg or 12 mg for those weighing ≥60 kg; anlotinib 12 mg orally once daily; apatinib 250 mg orally once daily; donafenib 0.2 g orally twice daily; or bevacizumab 15 mg/kg intravenously every 3 weeks. Concurrently, patients received at least one ICI via intravenous infusion every 3 weeks, including tislelizumab 200 mg, sintilimab 200 mg, camrelizumab 200 mg, atezolizumab 1200 mg, toripalimab 240 mg, or pembrolizumab 200 mg.

### Treatment response and safety assessment

Magnetic resonance imaging (MRI) or contrast-enhanced computed tomography (CT) were used to evaluate the tumor response in accordance with the mRECIST (21). Responses were categorized as complete response (CR), partial response (PR), stable disease (SD), or progressive disease (PD). The objective response rate (ORR) was defined as the proportion of patients achieving confirmed CR or PR, with the response maintained for 3–4 weeks after the last cycle of triple therapy. The disease control rate (DCR) was defined as the proportion of patients with CR, PR, or SD. Treatment safety was evaluated through documentation of treatment-related adverse events (TRAEs), which were graded in accordance with the National Cancer Institute Common Terminology Criteria for Adverse Events version 5.0 (NCI-CTCAE v5.0) (22).

### Conversion surgery

Notably, conversion surgery is defined as a post-treatment outcome rather than a baseline variable. It reflects the therapeutic response to triple therapy and is only applicable to a subset of patients who achieve tumor downstaging or functional liver reserve improvement. Eligibility for potentially curative hepatectomy was reassessed in accordance with Chinese guidelines (18). When patients met the criteria for resectability, conversion surgery was considered after multidisciplinary team discussion and after informed consent had been obtained. The procedure was performed at least 4 weeks after the last TACE cycle for eligible patients who provided informed consent. Bevacizumab was discontinued 6 weeks prior to hepatectomy, ICIs approximately 4 weeks prior, and TKIs 1–2 weeks prior. All hepatectomies were performed at high-volume hepatobiliary surgery centers by experienced hepatobiliary teams with more than 10 years of clinical experience in liver resection. The extent and method of liver resection in each patient were tailored based on the stage, location and size of tumors, quantitative liver function assessment, and patient’s physiological reserve. In cases of unsuccessful conversion treatment, TKIs and ICIs were continued until disease progression or the occurrence of intolerable adverse events.

### Study endpoints

After the initial cycle of combination treatment, all patients underwent close follow-up, starting with monthly appointments. Imaging investigations, laboratory testing, and physical examinations were all part of each follow-up evaluation.

OS, which is the period from the initial administration of triple therapy to the date of all-cause death or the final follow-up, was the main endpoint of this research. Secondary endpoints included PFS, ORR, DCR, and TRAEs. PFS was defined as the duration from the first dose of triple therapy to tumor progression (in line with mRECIST criteria), or death from any cause. Outcome data were collected during outpatient visits, hospital readmissions, or telephone follow-ups, with the final follow-up conducted on April 15, 2024.

### Statistical analysis

Frequencies and percentages were used to summarize categorical variables, and depending on the data distribution, Pearson’s chi-square test or Fisher’s exact test were used for between-group comparisons. Continuous variables were dichotomized according to clinically meaningful cutoff values. Variables demonstrating significant associations with OS in univariate analysis (P < 0.05) were incorporated into the multivariate Cox proportional hazards model, and backward stepwise elimination was utilized to identify independent prognostic factors. A prognostic scoring system was developed by weighting each independent prognostic factor based on its β coefficient derived from the final multivariate model. The predictive performance of the model was assessed by computing the area under the receiver operating characteristic curve (AUC). Kaplan-Meier survival curves were used to show risk stratification, and the log-rank test (two-sided α = 0.05) was used to compare strata. The prognostic scoring model was externally validated in an independent validation cohort to confirm its robustness. All statistical analyses were performed using R statistical software (version 4.5.1), and a two-tailed P value < 0.05 was considered statistically significant.

## Results

### Patients and characteristics

**Figure 1:**
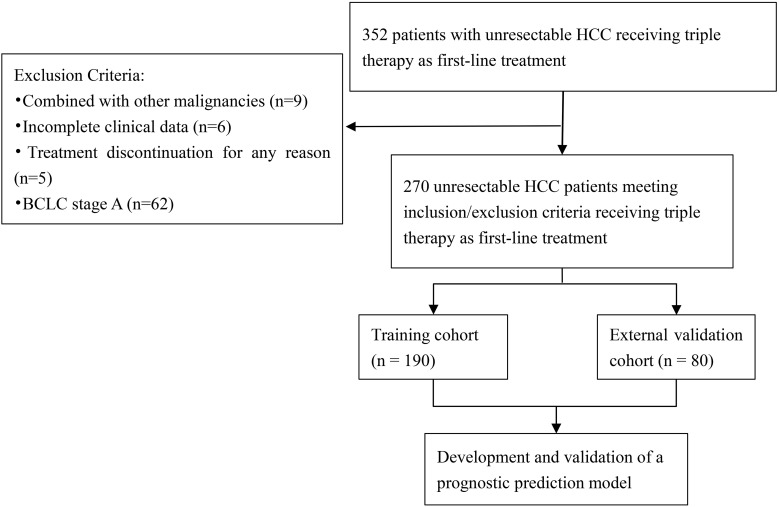
Flowchart of patient selection.

Following the application of inclusion and exclusion criteria, 270 eligible patients from an initial cohort of 352 patients with uHCC who received triple therapy as first-line treatment were ultimately included. These eligible patients were stratified into a training cohort (n=190) and an independent external validation cohort (n=80) based on the different participating medical centers. The detailed patient selection workflow is shown in **Figure 1**, and baseline demographic and clinical characteristics are summarized in **Table 1**. Both cohorts were predominantly male, accounting for 83.7% in the training cohort and 90.0% in the validation cohort, with hepatitis B virus (HBV) infection as the primary etiological factor. According to the BCLC staging system, most patients presented with advanced disease (BCLC stage C: 63.2% in training, 56.2% in validation). Furthermore, macrovascular invasion was evenly distributed between the two cohorts, with a prevalence of 50.0% in each, while extrahepatic metastasis was present in 24.2% and 18.8% of patients in the two groups. Conversion therapy followed by surgical resection was performed in 31.1% and 41.2% of patients in the training and validation cohorts, respectively. Laboratory findings showed that albumin-bilirubin (ALBI) grade 2 or 3 was present in 66.8% of the training cohort and 60.0% of the validation cohort, and elevated alpha-fetoprotein (AFP) levels (≥400 ng/mL) were documented in 45.8% and 52.5%, respectively. No statistically significant differences were observed across all key variables between the training and validation cohorts, confirming the comparability of the two cohorts for subsequent model development and validation.

**Table 1:** Baseline demographic and clinical characteristics of included patients.

Variables, n (%)	Training cohort (n=190)	Validation cohort (n=80)	P value
Age, mean years ± SD	52.68 ± 10.79	50.00 ± 10.91	0.064
Age			0.156
<55 years	109 (57.4)	54 (67.5)	
≥55 years	81 (42.6)	26 (32.5)	
Sex			0.247
Female	31 (16.3)	8 (10.0)	
Male	159 (83.7)	72 (90.0)	
Alcohol			0.735
No	137 (72.1)	60 (75.0)	
Yes	53 (27.9)	20 (25.0)	
Child-Pugh class			0.069
A	152 (80.0)	72 (90.0)	
B	38 (20.0)	8 (10.0)	
ECOG-PS			0.960
0	140 (73.7)	58 (72.5)	
1	50 (26.3)	22 (27.5)	
BCLC stage			0.354
B	70 (36.8)	35 (43.8)	
C	120 (63.2)	45 (56.2)	
HBV infection			0.548
No	36 (18.9)	12 (15.0)	
Yes	154 (81.1)	68 (85.0)	
TBil			0.727
<17 μmol/L	127 (66.8)	51 (63.7)	
≥17 μmol/L	63 (33.2)	29 (36.2)	
Albumin			0.145
<35 g/L	56 (29.5)	16 (20.0)	
≥35 g/L	134 (70.5)	64 (80.0)	
ALBI grade			0.350
1	63 (33.2)	32 (40.0)	
2 and 3	127 (66.8)	48 (60.0)	
ALT			0.063
<40 IU/L	94 (49.5)	29 (36.2)	
≥40 IU/L	96 (50.5)	51 (63.7)	
AST			0.145
<40 IU/L	56 (29.5)	16 (20.0)	
≥40 IU/L	134 (70.5)	64 (80.0)	
AFP			0.382
<400 ng/mL	103 (54.2)	38 (47.5)	
≥400 ng/mL	87 (45.8)	42 (52.5)	
Maximum tumor size			0.388
<8cm	74 (38.9)	26 (32.5)	
≥8cm	116 (61.1)	54 (67.5)	
Tumor number			0.52
Single	59 (31.1)	21 (26.2)	
Multiple	131 (68.9)	59 (73.8)	
Macrovascular invasion			>0.999
No	95 (50.0)	40 (50.0)	
Yes	95 (50.0)	40 (50.0)	
Extrahepatic metastasis			0.412
No	144 (75.8)	65 (81.2)	
Yes	46 (24.2)	15 (18.8)	
Surgery			0.141
No	131 (68.9)	47 (58.8)	
Yes	59 (31.1)	33 (41.2)	

SD, Standard Deviation; BCLC, Barcelona Clinic Liver Cancer; ECOG-PS, Eastern Cooperative Oncology Group performance status; HBV, hepatitis B virus; ALBI, albumin-bilirubin; ALT, alanine aminotransferase; AST, aspartate aminotransferase; AFP, alpha-fetoprotein.

### Treatment response and safety assessment

Tumor response in the training cohort was assessed in accordance with the mRECIST, with findings summarized in **Table 2**. Among the 190 patients, 20.5% attained CR, 27.4% achieved PR, 15.3% exhibited SD, and 36.8% developed PD. The ORR was 47.9% (91/190), while the DCR reached 63.2% (120/190).

**Table 2:** Tumor responses per mRECIST in the training cohort.

Best response, n (%)	Training cohort (n=190)
Complete response	39(20.5)
Partial response	52(27.4)
Stable disease	29(15.3)
Progressive disease	70(36.8)
Objective response rate	91(47.9)
Disease control rate	120(63.2)

The safety profile of triple therapy was evaluated based on TRAEs in the training cohort, as presented in **Table 3**. TRAEs of any grade were documented in 159 of 190 patients (83.7%), with Grade 3–4 events occurring in 35.8% of cases. The most common TRAEs predominantly comprised TACE-related events (consistent with post-embolization syndrome and hepatic injury), including abnormal liver function (55.3%), abdominal pain (43.2%), nausea (35.3%), vomiting (31.6%), and fever (24.7%), as well as systemic therapy-related events (TKIs/ICIs), such as hypertension (34.2%), anemia (31.1%), hypoalbuminemia (31.1%), hand-foot syndrome (27.4%), and rash (24.2%). No treatment-related deaths were reported. Despite the relatively high incidence of severe adverse events associated with triple therapy, the regimen remained well-tolerated, as most TRAEs were mild-to-moderate and clinically manageable.

**Table 3:** Treatment-related adverse events in training cohort.

Adverse event, n (%)	Any grade	Grade 1-2	Grade 3-4
Total	159 (83.7)	91 (47.9)	68 (35.8)
Abnormal liver function	105 (55.3)	65 (34.2)	40 (21.1)
Abdominal pain	82 (43.2)	55 (28.9)	27 (14.2)
Nausea	67 (35.3)	51 (26.8)	16 (8.4)
Hypertension	65 (34.2)	60 (31.6)	5 (2.6)
Vomiting	60 (31.6)	25 (13.2)	18 (9.5)
Anemia	59 (31.1)	36 (19.0)	13 (6.8)
Hypoalbuminemia	59 (31.1)	31 (16.3)	9 (4.7)
Hand-foot syndrome	52 (27.4)	31 (16.3)	21 (11.1)
Fever	47 (24.7)	42 (22.1)	5 (2.6)
Rash	46 (24.2)	24 (12.6)	22 (11.6)
Decreased appetite	40 (21.1)	37 (19.5)	3 (1.6)
Decreased WBC count	35 (18.4)	32 (16.8)	3 (1.6)
Fatigue	30 (15.8)	26 (13.7)	4 (2.1)
Decreased platelet count	28 (14.7)	21 (11.1)	7 (3.7)
Diarrhea	22 (11.6)	15 (7.9)	7 (3.7)
Hypothyroidism	15 (7.9)	13 (6.8)	2 (1.1)

WBC, white blood cell.

### Survival analysis

**Figure 2:**
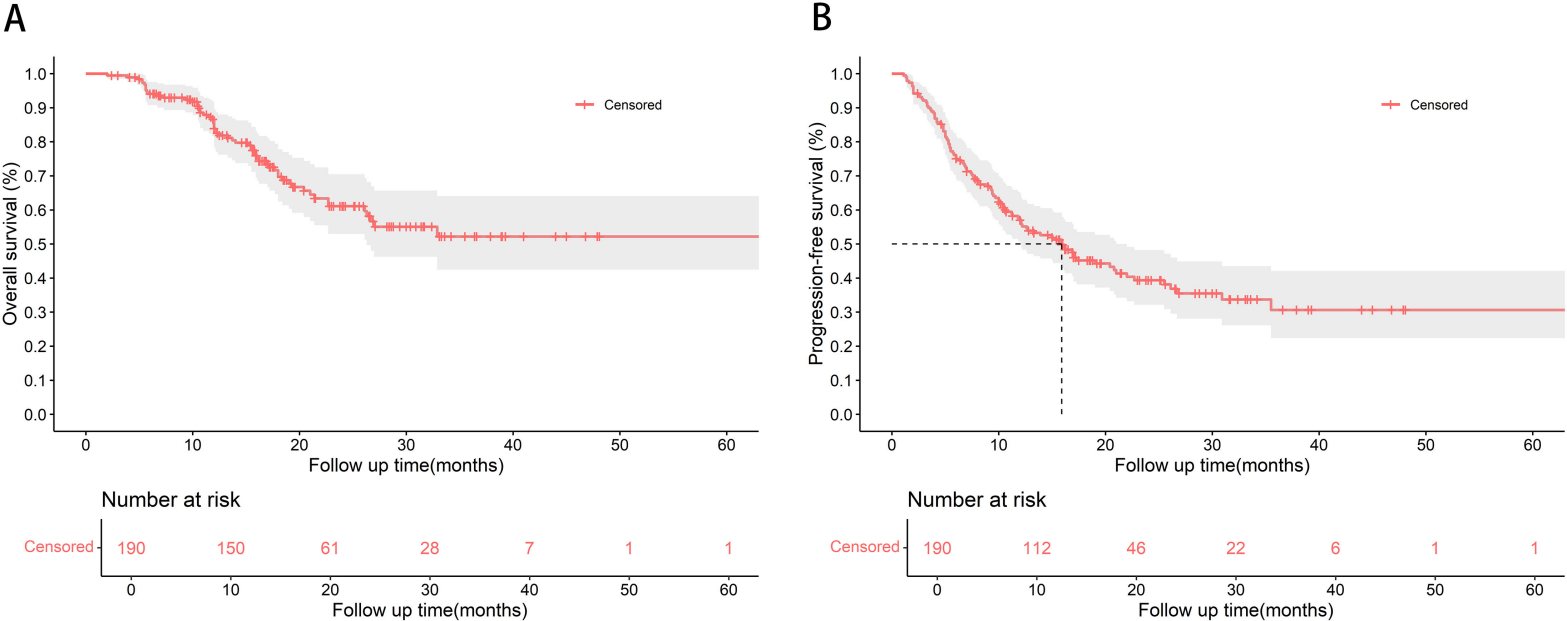
Kaplan-Meier curves for **(A)** overall survival and **(B)** progression-free survival for patients in the training cohort. Median overall survival was not reached.

With a median follow-up duration of 19.5 months (95% confidence interval [CI]: 17.3–24.1), 58 patients (30.5%) in the training cohort died during the follow-up period. Median OS was not reached, and the 1-year, 2-year, and 3-year OS rates were 83.9%, 61.1%, and 52.2%, respectively (**Figure 2A**). Median PFS was 15.9 months (95% CI: 12.1–22.0), with 1-year, 2-year, and 3-year PFS rates of 57.0%, 39.4%, and 30.7%, respectively (**Figure 2B**).

### Independent prognostic factors

Univariate and multivariate Cox proportional hazards regression analyses were carried out in order to identify robust predictors of OS in patients with uHCC receiving triple treatment (**Table 4**). In the univariate analysis, several clinical, laboratory, and tumor-related variables were significantly associated with inferior OS, including BCLC stage C, AST ≥40 IU/L, ALBI grade 2 and 3, AFP ≥400 ng/mL, maximum tumor size ≥8 cm, presence of EHM, and absence of conversion surgery. Variables that were statistically significant in the univariate analysis were included in the multivariate Cox regression model in order to eliminate potential confounding effects. The results confirmed that ALBI grade 2-3, AFP ≥400 ng/mL, maximum tumor size ≥8 cm, presence of extrahepatic metastasis, and no conversion surgery were independent prognostic factors for poor OS in this patient population.

**Table 4:** Univariate and multivariate cox regression analyses for OS in the training cohort.

Variables	Univariate analysis		Multivariate analysis	
	HR (95% CI)	P	HR (95% CI)	P
Sex (Male/Female)	1.12 (0.55–2.29)	0.747		
Age (≥55 years/<55years)	1.03 (0.61–1.74)	0.915		
Alcohol (Yes/No)	0.64 (0.33–1.23)	0.181		
HBV infection (Yes/No)	1.22 (0.58–2.58)	0.596		
BCLC stage (C/B)	2.81 (1.46–5.42)	**0.002**	1.03 (0.49–2.18)	0.932
Child-Pugh class (B/A)	1.52 (0.83–2.79)	0.171		
ALT(≥40 IU/L/<40 IU/L)	1.08 (0.64–1.80)	0.777		
AST(≥40 IU/L/<40 IU/L)	2.07 (1.09–3.91)	**0.025**	1.08 (0.55–2.13)	0.828
TBil (≥17 μmol/L/<17 μmol/L)	1.20 (0.70–2.06)	0.510		
ALBI grade (2 and 3/1)	2.92 (1.51–5.65)	**0.001**	2.82 (1.43–5.57)	**0.003**
AFP (≥400 ng/mL/<400 ng/mL)	2.42 (1.41–4.13)	**0.001**	1.81 (1.03–3.17)	**0.038**
Macrovascular invasion (Yes/No)	1.42 (0.84–2.39)	0.188		
Maximum tumor size (≥8 cm/<8 cm)	2.47 (1.35–4.52)	**0.003**	2.62 (1.38–4.99)	**0.003**
Tumor number (Multiple/Single)	1.44 (0.81–2.53)	0.212		
Extrahepatic metastasis (Yes/No)	3.62 (2.15–6.11)	**<0.001**	2.23 (1.21–4.10)	**0.010**
Surgery (No/Yes)	4.76 (2.16–10.5)	**<0.001**	3.89 (1.69–8.96)	**0.001**

HBV, hepatitis B virus; BCLC, Barcelona Clinic Liver Cancer; ALT, alanine aminotransferase; AST, aspartate aminotransferase; TBil, total bilirubin; ALBI, albumin-bilirubin;AFP, alpha-fetoprotein. Bold values denote statistically significant differences (p < 0.05).

### Development and validation of prognostic scoring model

Drawing on the independent predictors ascertained via multivariate Cox regression analysis, we developed a scoring model to stratify the prognosis of uHCC patients treated with triple therapy, named the SAGES score. This score integrates five key variables, in which each letter in “SAGES” corresponds to a key prognostic variable: S (maximum tumor size), A (AFP), G (ALBI grade), E (EHM), and S (conversion surgery).

The β coefficients of these five independent prognostic factors derived from the multivariate model were used to assign integer scores proportionally: maximum tumor size ≥8 cm (β=0.9842) was assigned 2 points, AFP ≥400 ng/mL (β=0.6016) 1 point, ALBI grade 2 and 3 (β=1.0552) 2 points, presence of EHM (β=0.8123) 2 points, and absence of conversion surgery (β=1.3729) 3 points (**Table 5**). The total SAGES score ranged from 0 to 10, and patients were categorized into three groups based on the prognosis: low-risk (0–3 points), intermediate-risk (4–7 points), and high-risk (8–10 points).

**Table 5:** Calculation and classification of the SAGES score.

Variables	β coefficient	HR (95% CI)	Scores
Maximum tumor size	0.9842		
< 8 cm		1 [Reference]	0
≥ 8 cm		2.68 (1.44-4.98)	2
Surgery	1.3729		
Yes		1 [Reference]	0
No		3.95 (1.73-9.01)	3
Extrahepatic metastasis	0.8123		
Absent		1 [Reference]	0
Present		2.25 (1.29-3.94)	2
AFP	0.6016		
< 400 ng/mL		1 [Reference]	0
≥ 400 ng/mL		1.83 (1.05-3.18)	1
ALBI grade	1.0552		
1		1 [Reference]	0
2 and 3		2.87 (1.48-5.58)	2
Model classification			
Low risk			0-3
Intermediate risk			4-7
High risk			8-10

HR, hazard ratio; CI, confidence interval; AFP, alpha-fetoprotein; ALBI, albumin-bilirubin.

**Figure 3:**
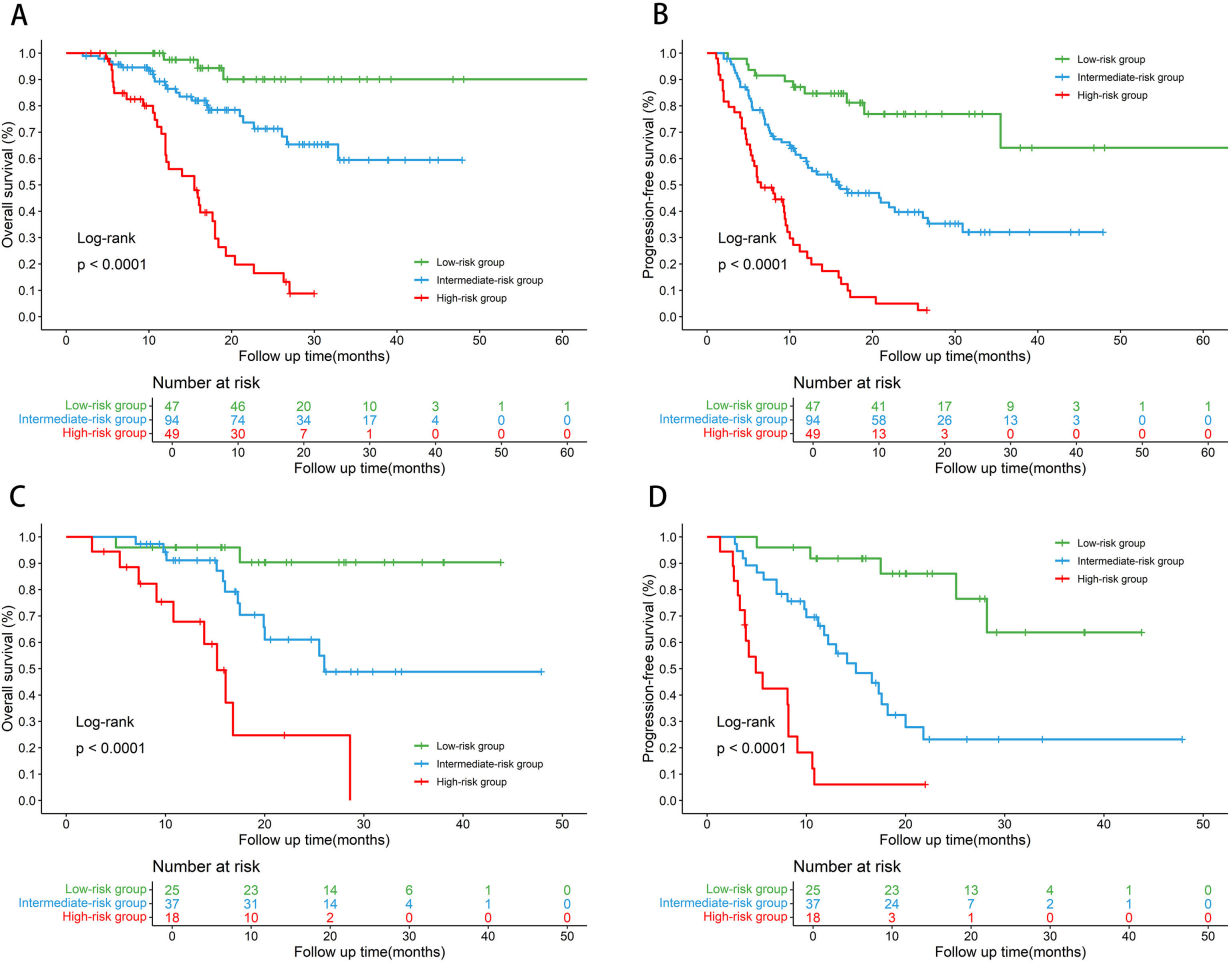
Kaplan-Meier curves according to SAGES score. **(A)** overall survival and **(B)** progression-free survival according to the SAGES score in the training cohort; **(C)** overall survival and **(D)** progression-free survival according to the SAGES score in the validation cohort.

In the training cohort, the SAGES score effectively stratified patients into three distinct risk strata with significantly divergent survival outcomes. The low-risk group achieved the most favorable prognosis, followed by the intermediate-risk group, while the high-risk group exhibited a substantially inferior survival profile. Specifically, the 2-year OS rates were 90.07% in the low-risk group, 71.32% in the intermediate-risk group, and 16.48% in the high-risk group (shown in **Figure 3A**). Furthermore, the SAGES score also significantly stratified PFS (shown in **Figure 3B**), with 2-year PFS rates of 76.91%, 39.70%, and 4.95% in the three risk groups, respectively (p < 0.001). These findings highlight the robust discriminative ability of the SAGES score for both OS and PFS.

The prognostic value of this model was further validated in an independent external cohort. Consistent with the findings in the training cohort, the SAGES score successfully stratified patients in the validation cohort into low-, intermediate-, and high-risk groups, with significant differences in both OS and PFS (both p < 0.0001, shown in **Figures 3C, D**). The 2-year OS rates were 90.4% (low-risk), 61.04% (intermediate-risk), and 24.73% (high-risk). Moreover, the 2-year PFS rates were 86.09% for the low-risk group and 23.18% for the intermediate-risk group. Collectively, these results confirm the robust and reproducible prognostic performance of the SAGES score across independent datasets.

### Performance of the SAGES score

**Figure 4:**
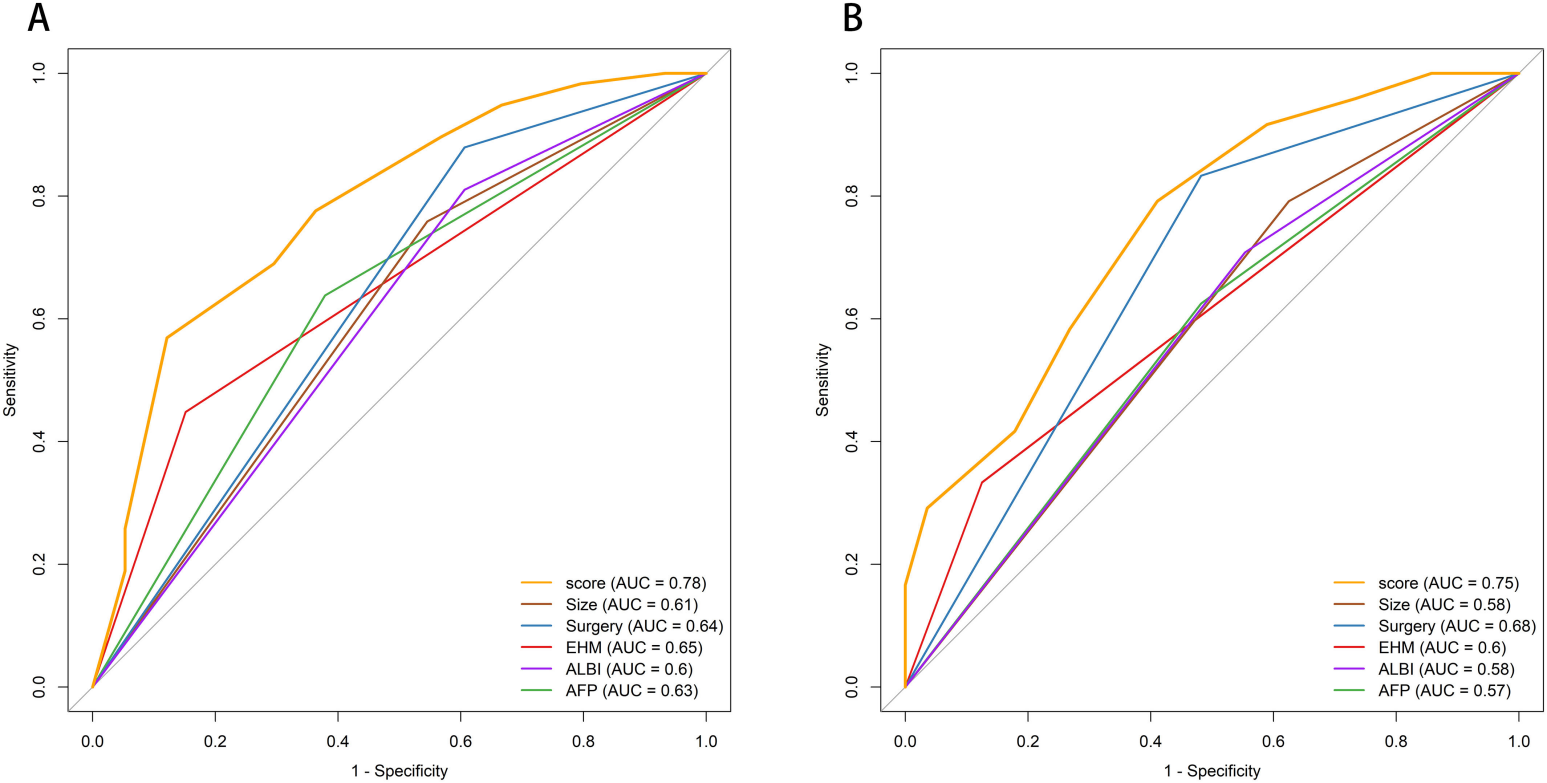
Comparison of ROC curves for overall survival in the **(A)** training cohort and **(B)** validation cohort. ROC, receiver operating characteristic curve; AUC, area under the receiver operating characteristic curve.

The discriminative ability of the SAGES score for OS was evaluated via receiver operating characteristic curve (ROC) curves in the training and external validation cohorts (shown in **Figure 4**).

In the training cohort, the SAGES score exhibited robust discriminative ability for OS prediction, with an AUC of 0.78 (95% CI: 0.71-0.85). By contrast, its individual component prognostic factors showed markedly weaker discriminative ability: maximum tumor size (AUC = 0.61, 95% CI: 0.54-0.68), conversion surgery (AUC = 0.64, 95% CI: 0.58-0.70), EHM (AUC = 0.65, 95% CI: 0.58-0.72), ALBI grade (AUC = 0.60, 95% CI: 0.54-0.67), and AFP level (AUC = 0.63, 95% CI: 0.55-0.71). Consistently, the SAGES score retained satisfactory discriminative capacity in the validation cohort (AUC = 0.75, 95% CI: 0.64-0.86), while individual factors showed weaker performance (AUC range: 0.57–0.68). The consistent superiority of the SAGES score over its individual components across both cohorts confirms the integrated model’s enhanced ability to distinguish OS outcomes, supporting its reliability for clinical prognostic stratification.

## Discussion

The present multicenter retrospective study investigated the efficacy and safety of triple therapy as first-line treatment for uHCC and developed a novel prognostic scoring system to stratify patient outcomes. Our findings demonstrated that this triple therapy regimen yields encouraging clinical benefits, with an ORR of 47.9% and a DCR of 63.2% per mRECIST criteria. Notably, the median OS was not reached during a median follow-up of 19.5 months, with a 3-year OS rate of 52.2%, and the median PFS was 15.9 months. These survival outcomes are comparable to those reported in previous triple therapy studies focusing on single TKI agents, highlighting the feasibility of incorporating multiple TKIs into the combination regimen for patients who may require medication adjustments due to adverse events or other clinical considerations (23–25).

The favorable antitumor activity of this triple therapy was slightly superior to that reported in previous studies using systemic therapy alone, which is mainly ascribed to the synergistic mechanisms among its components (26, 27). TACE serves as a foundational locoregional treatment by inducing tumor necrosis through targeted arterial embolization and chemotherapy, which not only reduces tumor burden directly but also facilitates the release of tumor-associated antigens, thereby enhancing the antitumor immune response triggered by ICIs (28–30). TKIs, including lenvatinib and other agents used in this study, exert anti-angiogenic effects by inhibiting vascular endothelial growth factor receptors, thereby disrupting the tumor vasculature and reversing the immunosuppressive tumor microenvironment (31–33). This vascular normalization and immune modulation further sensitize tumors to ICIs, which block the immune checkpoint pathway to reactivate exhausted T cells (34, 35). The integration of multiple TKIs in our study addresses a critical clinical scenario: patients may experience variable tolerability to different TKIs, and our results confirm that switching or selecting alternative TKIs does not impair the overall efficacy of the triple therapy, expanding the applicability of this regimen in real-world clinical practice. Notably, a considerable proportion of patients who achieved tumor downstaging after triple therapy subsequently underwent curative-intent hepatectomy for initially unresectable disease. This subgroup contributed to remarkably improved long-term survival, which disproportionately elevated the survival outcomes of the entire study population.

Safety remains a key consideration in combination therapy, especially for patients with underlying liver dysfunction. Only patients with an ECOG-PS score of 0 to 1 and a Child-Pugh score of less than 8 were enrolled in the study, as hepatic functional reserve can affect a patient’s medication tolerance and the efficacy of the treatment. The most common TRAEs, including abnormal liver function and hypertension, were in line with the established safety profiles of TACE, TKIs, and ICIs. The abnormal liver function, especially transient elevation of transaminases, is likely attributable to TACE-induced ischemic injury to hepatocytes, which has been previously reported as a potential surrogate marker of treatment response to TACE (36). Grade 3–4 TRAEs were relatively more frequent with triple therapy in our study. Nevertheless, the regimen yielded substantial clinical benefits. It markedly improved survival outcomes, enhances tumor response rates, and delivers survival benefits that outweigh the risks associated with increased adverse events. Thus, triple therapy is deemed clinically justified in this patient population. Consistent with trends from studies including EMERALD-1 and LEAP-012, which reported increased adverse events but significant survival advantages with TACE combined with immunotherapy and TKIs, our findings further support the clinical value of combination therapy in patients with definite benefits, even with a modest increase in adverse events (37, 38). Close surveillance of liver function and TRAEs throughout treatment not only ensures patient safety but also enables timely dose adjustments or supportive care, thereby improving treatment compliance and prolonging patients’ survival.

A major novelty of our research is the development and external validation of the SAGES score, a prognostic model integrating five independent factors of OS. Each component of the SAGES score is clinically meaningful and supported by existing evidence. Maximum tumor size ≥ 8 cm and EHM are established markers of advanced tumor burden, indicating increased invasiveness and metastatic potential, which are consistent with previous findings that tumor burden is a key determinant of survival in uHCC (23, 31, 39–41). AFP, a classic blood biomarker for HCC, suppresses antitumor immunity in addition to being associated with tumor aggressiveness and proliferation, making it a robust prognostic indicator for uHCC patients receiving systemic or combination therapy (23, 40). The ALBI grade is a validated metric for evaluating hepatic functional reserve. Patients with ALBI grade 2–3 exhibit impaired liver function, which is associated with reduced treatment tolerance and poorer outcomes (39, 42). Notably, the inclusion of conversion surgery as a prognostic factor underscores the clinical value of conversion therapy. Patients who achieved resectability after triple therapy usually have a better prognosis, highlighting the potential curative role of surgical resection in selected patients (43, 44). Importantly, we emphasize that conversion surgery is a post-treatment outcome (dependent on triple therapy response) rather than a pre-treatment baseline variable, which determines the core application scenario of the SAGES score: it is not a tool for pre-treatment patient screening, but rather for early prognostication after triple therapy initiation. For clinicians, this score may help identify uHCC patients who can achieve the most favorable benefit from triple therapy. Meanwhile, it also indicates that patients stratified as high risk may derive limited benefit from standard triple therapy alone. For this population, we may need to explore other novel combination regimens, such as HAIC, radiotherapy, or other novel combination regimens, to facilitate individualized precision treatment.

There are a number of prognostic scoring models available for HCC patients receiving systemic or locoregional treatments, but none for uHCC patients administered triple therapy of TACE plus TKIs and ICIs (23, 45–47). Zeng et al. created the TAE score, Scheiner et al. developed the CRAFITY score and Zhang et al. created the PPRD score to predict the survival of HCC patients receiving different treatments (23, 45, 46). In our study, the SAGES score demonstrates robust discriminative capability, with a key advantage residing in the integration of ALBI grade and conversion surgery, which effectively addresses critical limitations of existing prognostic tools. Additionally, the score is easily calculable using routine laboratory parameters, enabling early risk stratification to guide personalized treatment: high-risk patients may benefit from more intensive therapy or tighter monitoring, while low-risk patients can receive standard triple therapy without unnecessary escalation. Most prior triple therapy studies for uHCC focused on single TKIs and lacked regimen-specific prognostic models. Our study extends this evidence by incorporating multiple TKIs and validating the SAGES score through a multicenter design with external validation, thereby enhancing its generalizability and potential as a clinical decision-making tool.

Despite these strengths, several limitations of this study should be acknowledged. First, as a retrospective study, this study has inherent selection bias that cannot be completely eliminated, and its non-randomized design limits the ability to compare the efficacy of different TKIs or the triple therapy with other therapeutic regimens. Second, while the median follow-up duration of 19.5 months is adequate for evaluating short- and mid-term outcomes, longer follow-up is needed to confirm the long-term survival benefit of triple therapy and the prognostic stability of the SAGES score. Third, the study population was predominantly composed of patients with HBV infection, which may limit the applicability of the SAGES score to patients with other etiologies (e.g., hepatitis C virus, non-alcoholic steatohepatitis). Fourth, the inclusion of various TKIs and ICIs inherently introduces heterogeneity in treatment responses. Due to the limited overall sample size, we were unable to conduct robust subgroup analyses based on specific drug combinations. The small number of patients within individual regimen subgroups would result in underpowered statistical comparisons, thereby compromising the reliability of any subgroup-specific conclusions. Fifth, this study was conducted across 20 participating centers, which could not completely eliminate the differences between TACE operations. Despite the utilization of standardized key technical parameters and experienced operators, subtle variations may inevitably exist in interventional techniques, assessment of embolic endpoints, and intra-procedural dose adjustments. These procedural variations could potentially impact tumor necrosis rates, liver function recovery, and subsequent treatment responses, introducing certain bias into our findings. Sixth, as conversion surgery is evaluated as a post-treatment outcome, its incorporation inherently restricts the SAGES score to serving as an early in-treatment prognostic tool rather than a baseline pre-treatment selection criterion.

Future studies should address these limitations by conducting large-scale, multicenter prospective trials to validate the SAGES score and compare the efficacy of triple therapy with different TKI combinations or other standard regimens. Additionally, exploring the predictive value of the SAGES score in combination with novel biomarkers (e.g., immune checkpoint molecules, circulating tumor DNA) may further improve prognostic accuracy. Further research is also needed to optimize treatment strategies for high-risk patients identified by the SAGES score, such as investigating the role of neoadjuvant triple therapy or combination with other targeted agents.

## Conclusion

In conclusion, the present study demonstrates that triple therapy combining TACE with multiple TKIs and ICIs is effective and well-tolerated as first-line treatment for uHCC. The SAGES score exhibits robust discriminative power and generalizability, enabling clinicians to identify optimal candidates for continued triple therapy and to guide personalized treatment decisions. These findings contribute to the optimization of multimodal therapy for uHCC and emphasize the significance of prognostic stratification in improving patient outcomes.

## Data Availability

The raw data supporting the conclusions of this article will be made available by the authors, without undue reservation.
